# The impact of short-term reduction in hepatic triglycerides on vascular health in adults with or at risk of metabolic dysfunction-associated steatotic liver disease

**DOI:** 10.1371/journal.pone.0345338

**Published:** 2026-08-13

**Authors:** Tianyu Wang, Isabelle Gallagher, Jessica Park, Yanrong Li, Marie Caillaud, Taha A. Alhalimi, Lin-Sheng Chen, Natalie N. McLaurin, Jack Knight-Scott, Jeffrey D. Browning, Andreana P. Haley, Hirofumi Tanaka

**Affiliations:** 1 Department of Kinesiology and Health Education, The University of Texas at Austin, Austin, Texas, United States of America; 2 Department of Psychology, The University of Texas at Austin, Austin, Texas, United States of America; 3 Centre de Recherche, The Centre Hospitalier Universitaire Sainte-Justine, Montréal, Québec, Canada; 4 Department of Radiology, Children’s Healthcare of Atlanta, Atlanta, GeorgiaUnited States of America; 5 Department of Clinical Nutrition, University of Texas Southwestern Medical Center, Dallas, Texas, United States of America; 6 Biomedical Imaging Center, The University of Texas at Austin, Austin, Texas, United States of America; 7 Faculty of Sport Science, Chulalongkorn University, Bangkok, Thailand; University of Montenegro-Faculty of Medicine, MONTENEGRO

## Abstract

**Background:**

Metabolic dysfunction-associated steatotic liver disease (MASLD) increases cardiovascular risk, but the vascular benefits of reducing hepatic triglycerides remain unclear.

**Aim:**

To determine whether short-term diet-induced reductions in hepatic triglycerides improve arterial stiffness and endothelial function.

**Methods:**

In a two-week, two-arm pre-post dietary intervention trial, adults with MASLD or elevated risk were assigned to either a low-carbohydrate (<30 g carbohydrate/day, n = 24) or low-calorie diet (~1,200−1,500 kcal/day, n = 23). Hepatic triglyceride content was assessed using proton density fat fraction magnetic resonance imaging. Vascular assessments included arterial stiffness as assessed by pulse wave velocity (PWV), and endothelial function via brachial artery flow-mediated dilation (FMD). The Framingham Risk Score (FRS) was evaluated pre- and post-intervention.

**Results:**

At baseline, 31 of 47 participants had MASLD. In the total sample, hepatic triglyceride content decreased by 15% relative to baseline (p < 0.001), with no significant differences between the two diet groups. Brachial-ankle PWV decreased by 41 cm/s (p = 0.001). FRS decreased (p < 0.001) and FMD (p = 0.030) increased by ~1.0% in absolute terms. Reductions in hepatic triglyceride content were positively correlated with changes in heart rate at rest (r = 0.49, p = 0.001), even after the adjustment of age and body weight change (r = 0.35, p = 0.021).

**Conclusions:**

Vascular function improvements and cardiovascular risk reductions occurred along with hepatic triglyceride reduction following a two-week dietary intervention. Changes in hepatic triglyceride content were positively correlated with the corresponding changes in heart rate but not with measures of vascular function. (ClinicalTrials.gov, NCT05216796).

## Introduction

Metabolic dysfunction-associated steatotic liver disease (MASLD) is the most common chronic liver disease globally, affecting 38% of adults and projected to increase to 55% by 2035 [1]. It is characterized by triglyceride accumulation in >5% of hepatocytes with at least one metabolic syndrome component and limited alcohol consumption [2]. Beyond its hepatic manifestation, MASLD is now recognized as a multi-system condition with substantial implications for cardiovascular health [3]. Notably, hepatic triglyceride accumulation has been proposed as both a marker and a potential mediator of vascular impairments such as endothelial dysfunction [4] and arterial stiffening [5]. Cardiovascular disease remains the leading cause of death in people with MASLD, accounting for approximately 40% of all-cause mortality in this population [3].

Lifestyle intervention targeting hepatic triglyceride reduction with weight loss is the first-line treatment for MASLD [6]. However, data are limited regarding the short-term vascular effects of these interventions and whether improvements in vascular function are directly linked to reductions in hepatic triglyceride. The primary aim of this study was to assess the impact of dietary intervention targeting hepatic triglyceride reduction on key subclinical markers of vascular function: arterial stiffness and endothelial function in individuals with MASLD. The secondary aim was to investigate how changes in hepatic triglyceride content relate to improvements in subclinical vascular measures. We hypothesized that the dietary intervention would lead to improvements in hepatic triglyceride content, arterial stiffness, and endothelial function. Furthermore, we posit that reductions in hepatic triglyceride content would be directly associated with improvements in subclinical vascular measures.

## Materials and Methods

### Participants

The participants were recruited from the Greater Austin metropolitan area, Texas, through flyers, newspaper advertisements, word-of-mouth, and social media. The recruitment period was from May 15, 2022, to August 3, 2024. The inclusion criteria include men or women of any race or ethnicity, at least 40 years of age and at high risk for MASLD based on the presence of cardiometabolic risk factors. Since fewer than 5% of adults with MASLD were aware that they had steatotic liver disease [7], we applied a likelihood-based screening approach to identify as many participants with MASLD as possible, while maintaining feasibility given our budget and the scheduling constraints for MRI assessments and dietary intervention delivery. Specifically, individuals were considered eligible if they (1) self-reported at least three metabolic syndrome components (abdominal obesity, elevated blood pressure, impaired fasting glucose, high triglyceride concentration, or low HDL cholesterol concentration) or (2) had a physician-confirmed or suspected diagnosis of MASLD. Due to the complexity of dietary and MRI-scan scheduling, we included individuals both with and without steatotic liver, as determined after a baseline liver MRI scan. The exclusion criteria included excess alcohol consumption (Alcohol Use Disorder Identification Test-Consumption score >5), other types of liver disease, participation in a dietary intervention within the past six months, currently following a specialized diet plan, or engagement in an exercise training program for weight loss, and contraindications to magnetic resonance imaging safety criteria. This study was approved by the Institutional Review Board at the University of Texas at Austin (STUDY00002189) and was registered at ClinicalTrial.gov (NCT05216796). This study is a secondary objective in the original registration trial. Electronic informed consent was obtained for all participants through DocuSign during the virtual screening visit. All data were collected at the University of Texas at Austin.

### Dietary intervention

Weight loss is the first-line therapeutic strategy for reducing hepatic triglyceride content in MASLD [8], and caloric restriction remains the most widely adopted dietary approach to achieve this goal. In line with current clinical guidelines and prior evidence, we designed two dietary interventions: a low-calorie diet (~70% of regular calorie intake, corresponding to ~1200 kcal/day for women and ~1500 kcal/day for men) and a carbohydrate-restricted diet (≤30 g carbohydrates/day). Both dietary strategies were adapted from a previous intervention study [9], which demonstrated that low-carbohydrate and low-calorie diets both reduce hepatic triglycerides to different extents. The primary goal is to induce a reduction in hepatic triglyceride levels and to examine the physiological consequences of changes in hepatic triglyceride levels. Accordingly, both dietary interventions that have been demonstrated to successfully reduce hepatic triglycerides in the short term were included. Comparisons between diets were not the primary focus of this study, and any subgroup analyses by diet were considered supplementary.

The study team randomly assigned eligible participants in a 1:1 ratio to either a low-carbohydrate diet or a low-calorie diet using block randomization, with five participants allocated to the low-carbohydrate diet followed by five to the low-calorie diet in each block. To prevent sudden dietary changes from affecting pre-intervention measures, participants followed a three-day weight maintenance diet based on their usual intake, which the team determined from a three-day diet recall. Nutritionist Pro^®^ (Axxya Systems) analyzed their dietary intake from the three-day diet records, assessing calorie, macronutrient, and micronutrient composition. A commercial meal service (Snap Kitchen, Snap Kitchen Investments, LLC, Austin, TX) provided standardized menu options according to participants’ food preferences and dietary intervention assignment. Low-carbohydrate meals were selected directly from these standardized options, whereas no predefined low-calorie category was available. Therefore, meals for the low-calorie group were selected by the study team from available options beyond the low-carbohydrate diet category and tailored to achieve ~70% of each participant’s habitual caloric intake. Participants were blinded to dietary intervention assignment until completion of the study. Study personnel were partially blinded, with two primary administrative members aware of the intervention allocation. Participants were instructed to maintain their habitual medication regimen throughout the intervention period. No participants reported any changes in their medication regimen during the two-week intervention. All participants reported adherence to the prescribed dietary intervention. However, no objective measures were used to quantify dietary adherence.

### Laboratory assessment

In the initial virtual laboratory visit, the research team conducted an online screening session to assess participants based on the inclusion and exclusion criteria. After confirming eligibility, participants provided informed consent and completed a comprehensive set of background questionnaires and medical history.

Prior to the cardiovascular visit, participants fasted for at least 12 hours and abstained from strenuous exercise, caffeine, and alcohol for 24 hours. Body weight and height were measured using an electronic scale and stadiometer with participants in light clothing and no shoes. Waist circumference was measured at the level of the umbilicus, and hip circumference at the widest part of the hips, using a non-elastic tape measure. Body composition, including body fat percentage, fat mass, and lean muscle mass, was measured through the bioimpedance method (Tanita MC-780U, Tokyo, Japan).

Arterial stiffness was assessed using pulse wave velocity (PWV) measurements with the Omron VP-1000plus device (Omron Healthcare, Kyoto, Japan) after a 10-minute supine rest in a quiet, temperature-controlled environment [10]. To assess stiffness in different arterial segments, carotid-femoral PWV and brachial-ankle PWV were measured. Measurements were taken at least twice until the difference between two PWV readings was less than 50 cm/s. Heart rate, systolic and diastolic blood pressure were recorded along with PWV. The average of two readings was used for data analysis. Carotid intima-media-thickness (IMT) was also evaluated using carotid artery ultrasonography with a linear-array transducer (L12-3) in B-mode imaging (Philips Affiniti 70, Amsterdam, Netherlands). Carotid IMT was measured on the far wall of the distal common carotid artery, approximately 1–2 cm proximal to the carotid bulb. It is a noninvasive marker of early atherosclerosis that reflects subclinical arterial wall remodeling before overt plaque formation [11]. The ultrasound images were analyzed using the Carotid Analyzer software (Medical Imaging Applications LLC, Iowa, USA).

Endothelium-dependent vasodilation was assessed using flow-mediated dilation (FMD). This method measures the relative change in brachial artery diameter in response to an ischemia-reperfusion protocol using ultrasound imaging with a linear-array transducer (L12-3) in B-mode (Philips Affiniti 70, Amsterdam, Netherlands). Blood flow occlusion was induced by inflating the cuff to 200 mmHg in the forearm for five minutes. After releasing the occlusion, the diameter was recorded to calculate FMD (%) as (peak diameter – baseline diameter)/ (baseline diameter) x 100% [12]. The distance from the upper border of the blood pressure cuff to the antecubital fossa and from the antecubital fossa to the ultrasound probe was measured and kept consistent between pre- and post-intervention assessments. All FMD assessments were performed by a single trained investigator with experience in vascular ultrasound imaging and FMD assessment. Ultrasound images were analyzed offline using automated edge-detection software by an investigator blinded to participant identity and study visit. All measurements were obtained following established guidelines using standardized acquisition and analysis procedures to minimize operator-related variability.

Venous blood samples were collected from the antecubital vein, and biochemical analyses were conducted by trained personnel. Inflammatory markers, including high-sensitive C-reactive protein (hs-CRP) and interferon gamma-induced protein 10 (IP-10), and insulin concentrations in serum were analyzed using commercial kits from R&D Health Systems (Minneapolis, MN, USA). Blood lipid profiles, including the concentrations of triglycerides, total cholesterol, high-density lipoprotein (HDL) cholesterol, low-density lipoprotein (LDL) cholesterol, and non-HDL cholesterol, along with fasting glucose concentrations, were measured using the Cholestech LDX analyzer (Alere, CA, USA). Insulin resistance was estimated using the Homeostatic Model Assessment of Insulin Resistance (HOMA-IR) equation [13]. Framingham Risk Score was calculated using a standard, sex-specific scoring system [14] in the lipid- and BMI-based models. The lipid-based model calculates risk using sex, age, systolic blood pressure, total cholesterol, HDL cholesterol, and three dichotomous variables: blood pressure medication use, smoking status, and diabetes mellitus diagnosis. The BMI-based model replaces total cholesterol and HDL cholesterol with BMI, while incorporating the same additional risk factors.

Hepatic triglyceride content was assessed using magnetic resonance imaging-proton density fat fraction (MRI-PDFF) on a 3T Siemens MRI scanner (Magnetom Vida, Siemens Healthineers, Erlangen, Germany) at the University of Texas at Austin Biomedical Imaging Center (RRID: SCR_021898). We used a specialized technique called high-speed T2-corrected multi-echo, which provides an accurate, non-invasive estimate of hepatic triglyceride by separating signals from fat and water [15]. A small imaging region (voxel) was carefully placed in the right lobe of the liver, avoiding major blood vessels or bile ducts to ensure precise measurements. The scanner then acquired multiple signals at different time points to correct for signal loss and improve accuracy. Hepatic triglyceride content was calculated as the percentage of the fat signal relative to the total fat and water signal. Individuals with hepatic fat content greater than 5%, along with at least one metabolic dysfunction criterion [2], are diagnosed with MASLD.

### Statistical analyses

The primary outcomes of this study include subclinical vascular measures and hepatic triglyceride content. The secondary outcomes are cardiometabolic and inflammation profiles. A priori power analysis (*α* = 0.05, power = 0.95, effect size = 0.3) indicated that a minimum of 40 participants were required to detect a significant change in hepatic triglyceride content based on previous literature [9], using G*Power 3·1.

Normality of distributions was assessed using the Shapiro–Wilk test. The extreme outliers were identified as those beyond 3 interquartile ranges defined by statistical analysis software (SPSS Statistics 29, IBM Corp., Armonk, NY). The outliers were removed for the correlation analyses. To assess overall changes following the dietary intervention, we conducted paired t-tests to evaluate alterations in primary subclinical atherosclerosis and cardiometabolic outcomes, as well as secondary demographic outcomes. We applied non-parametric tests for non-normally distributed variables: the Mann–Whitney U test for independent samples and the Wilcoxon signed-rank test for dependent samples. Categorical variables were analyzed using chi-square tests. The secondary two-way repeated measures Analysis of Variance (ANOVA) was performed to examine how dietary intervention and MASLD status influenced hepatic triglyceride content, vascular health, and associated risk factors after the intervention. Bonferroni correction was applied for post hoc comparisons.

We conducted both simple and partial correlation analyses to assess the relations between improvements in subclinical cardiovascular measures, hepatic triglyceride content, and metabolic variables, before and after adjusting for age and body weight change. This approach allowed us to identify both overall and age-body weight-independent associations, given that the outcome of interest is strongly influenced by age and body weight change. The changes were defined as post-intervention value minus the pre-intervention value. Pearson correlation (two-tailed) was used for normally distributed variables, and Spearman rank correlation (two-tailed) was used for non-normally distributed variables. To account for the influence of baseline values on the response, relative changes were calculated by dividing the difference between post- and pre-intervention values by the baseline value, which supplemented the correlation analysis. In addition, subgroup analyses were performed among participants with MASLD only to evaluate the pre-post change following the intervention and to examine the correlation between the changes in hepatic triglycerides and vascular measures.

All statistical analyses were conducted in SPSS Statistics 29 (IBM Corp., Armonk, NY). Graphs were generated using GraphPad Prism 10 (GraphPad Software, Boston, MA). Figures of partial correlation between two variables were plotted using their residuals obtained from the linear regression models. Each variable was regressed on the adjusted variables (i.e., age and body weight change). Aligning with the partial correlation approach, this procedure removes the variance explained by the age and body weight change, and the remaining residuals represent the variation in each variable that is independent of age and body weight change. The residuals of the variable from each regression model were extracted from SPSS and used to visualize the partial correlation results between the two variables.

## Results

**Fig 1:**
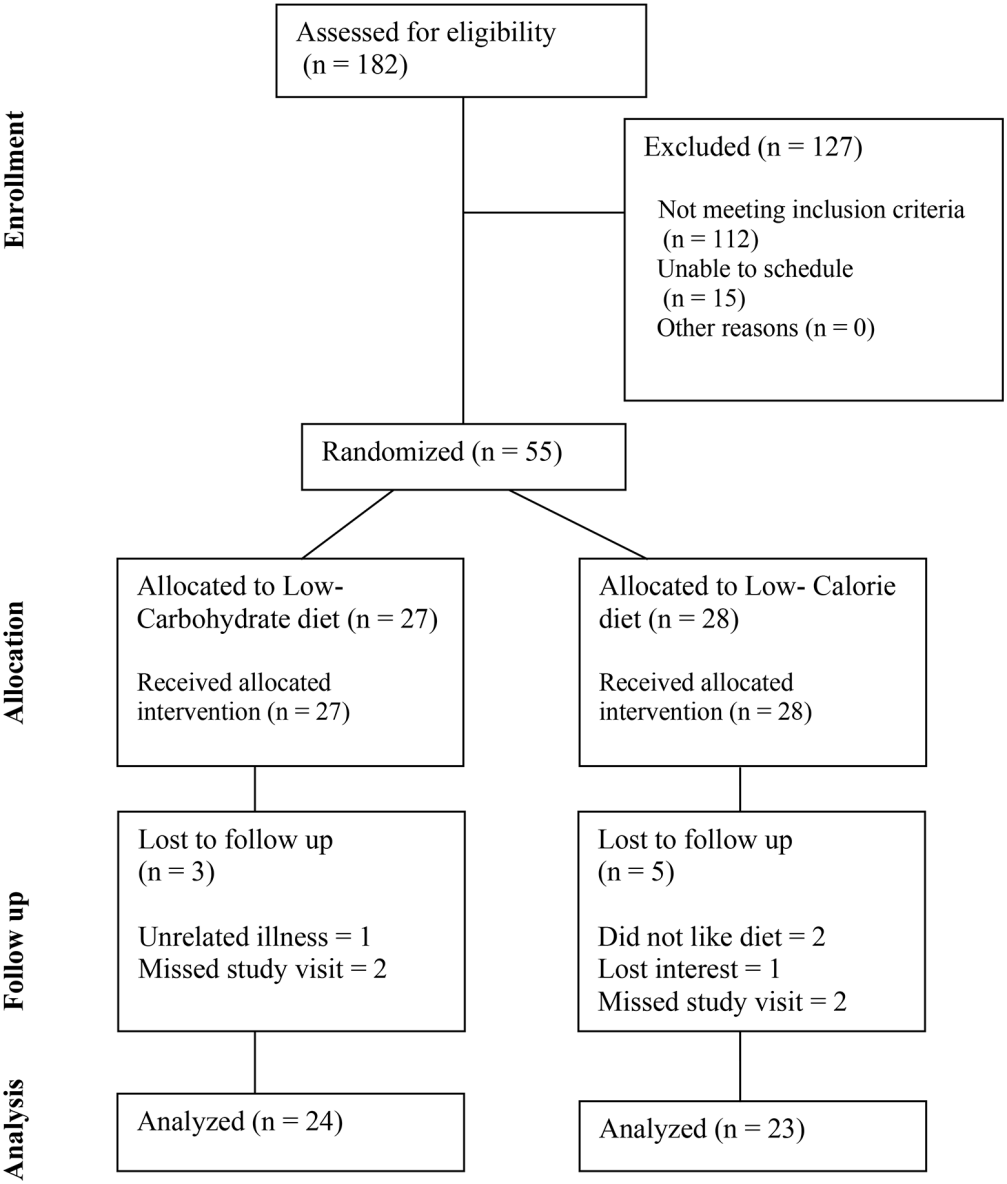
CONSORT diagram showing the flow of participants through each stage of the randomized trial.

A total of 47 participants completed the study (Fig 1). Participant characteristics for the overall sample and by MASLD status are presented in Table 1. No adverse events or unintended effects were reported in either group during the study period. Most participant characteristics were similar between the dietary groups (S1 Table). The mean age was 54 ± 9 years, and 81% were female. MASLD was present in 31 participants (66%). The distribution of the two diet types was similar between the MASLD and non-MASLD groups. The enzymatic analyses for insulin and inflammatory markers were completed in 41 participants. This is due to the unsuccessful venipuncture attempts or participant refusal for phlebotomy blood collection in at least one of their laboratory visits. FMD analysis was completed in 40 participants, with seven participants excluded due to inadequate image quality that precluded accurate detection of vessel diameter in one or more laboratory visits. The analysis strategy was intention-to-treat.

**Table 1:** Selected characteristics of participants with and without metabolic dysfunction-associated steatotic liver disease.

n (%) or means ± SD	Overall	Non-MASLD	MASLD	*p* value
Age (years)	54 ± 8	54 ± 9	54 ± 8	0.888
Female/Male	38/9 (81/19)	12/4 (75/25)	26/5 (84/16)	0.464
Race				0.089
White	29 (62)	7 (44)	22 (71)	
Black	7 (15)	3 (19)	4 (13)	
Asian	3 (6)	3 (19)	0 (0)	
Other	8 (17)	3 (19)	5 (16)	
Hepatic triglycerides (%)^b^	13.0 ± 10.7	2.7 ± 1.2	18.3 ± 9.4	**< 0.001**
Body weight (kg)	91.7 ± 19.4	80.9 ± 17.5	97.3 ± 18.5	**0.005**
Height (cm)	163.4 ± 7.6	162.9 ± 6.5	163.7 ± 8.2	**0.737**
Body mass index (kg/m^2^)	34.4 ± 7.1	30.5 ± 6.8	36.3 ± 6.6	**0.007**
Waist circumference (cm)	109 ± 15	100 ± 14	114 ± 13	**0.002**
Body fat percentage (%)	39 ± 8	35 ± 10	41 ± 6	**0.046**
Systolic BP (mmHg)	121 ± 11	118 ± 12	123 ± 11	0.114
Diastolic BP (mmHg)	70 ± 8	69 ± 8	71 ± 7	0.392
Total cholesterol (mmol/l)	4.8 ± 1.1	5.0 ± 1.2	4.7 ± 1.1	0.372
HDL cholesterol (mmol/l)^b^	1.1 ± 0.4	1.1 ± 0.4	1.1 ± 0.4	0.559
Non-HDL cholesterol (mmol/l)	3.7 ± 1.1	3.9 ± 1.3	3.6 ± 1.0	0.410
LDL cholesterol (mmol/l)^b^	3.1 ± 1.0	3.4 ± 1.2	2.9 ± 0.9	0.080
LDL/HDL cholesterol ratio	2.9 ± 1.4	3.5 ± 1.9	3.0 ± 1.0	0.292
Blood triglyceride (mmol/l)^b^	1.3 ± 0.6	1.1 ± 0.5	1.5 ± 0.6	**0.006**
Blood glucose (mmol/l)	5.4 ± 1.0	5.2 ± 0.9	5.6 ± 1.0	0.296
Serum insulin (µU/ml)^ab^	17.7 ± 15.4	7.5 ± 2.9	23.6 ± 16.6	**< 0.001**
HOMA-IR^ab^	4.4 ± 4.5	1.8 ± 0.9	5.8 ± 5.0	**< 0.001**
CRP (mg/l)^ab^	4.5 ± 4.5	3.9 ± 5.8	4.9 ± 3.6	**0.048**
Medications				
Blood pressure	13 (29)	3 (19)	10 (35)	0.223
Cholesterol	11 (24)	1 (6)	10 (35)	**0.035**
Blood glucose	8 (18)	0 (0)	8 (28)	**0.020**

^a^Overall sample size = 41, non-MASLD group = 14, and MASLD group = 27. ^b^ Non-normally distributed variable; comparisons were performed using the Mann-Whitney U test. All other continuous variables were analyzed using independent-sample t tests. Categorical variables were compared using the chi-square test. P-values represent comparisons between the non-MASLD and MASLD groups. Abbreviations: BP, blood pressure; HDL, high-density lipoprotein; LDL, low-density lipoprotein; HOMA-IR, homeostatic model assessment of insulin resistance; CRP, C-reactive protein.

Overall, both dietary interventions produced significant and comparable reductions in hepatic triglycerides, accompanied by improvements in most metabolic risk factors, with a more pronounced reduction in insulin levels and Framingham Risk Score based on the lipid model (S2 Table). Accordingly, the subsequent analyses were conducted using a pooled data sample.

**Fig 2:**
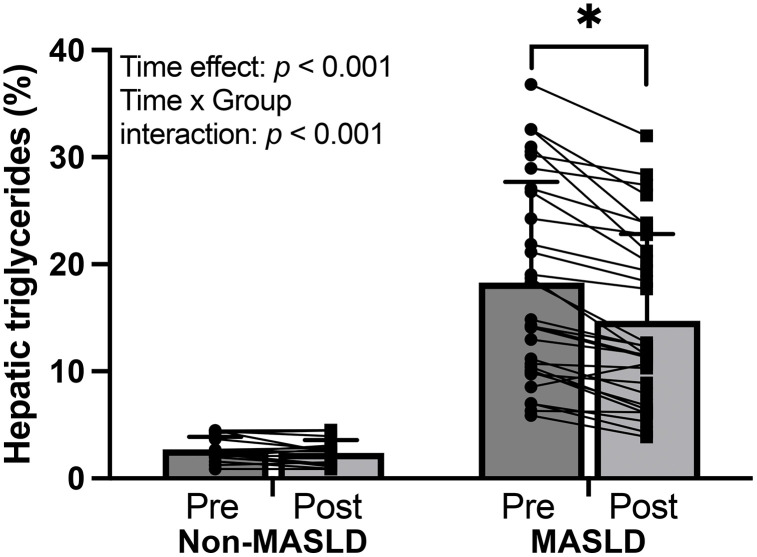
Reduction of hepatic triglyceride after the dietary interventions. Data were means ± SD. Comparisons were analyzed using a two-way ANOVA with one within-subject factor and one between-subject factor, followed by Bonferroni-adjusted post hoc comparisons. * *p* < 0.05.

In the total sample, hepatic triglyceride content, body weight, muscle mass, and adiposity metrics decreased significantly (Table 2). Body weight decreased by 2.9% (95% CI: [–3.5%, –2.3%]). Hepatic triglycerides decreased by 15% relative to baseline values (95% CI: [–21.4%, –9.4%]). When stratified by MASLD status, the reduction in hepatic triglyceride content (Two-way mixed ANOVA, *F*_*MASLD-time*_(1, 45) = 18.3, *p* < 0.001, partial η^2^ = 0.29) was more pronounced in participants with MASLD. Both variables were significantly higher at baseline in individuals with MASLD compared with those without MASLD (Fig 2).

**Table 2:** Changes in hepatic, metabolic, and cardiovascular outcomes from pre- to post-intervention in the total sample.

Mean ± SD	Pre (n = 47)	Post (n = 47)	Mean difference (95% CI)	*p* value
Hepatic triglycerides (%)^c^	12.6 ± 10.5	10.4 ± 8.8	**−2.2 (−2.9, −1.4)**	**< 0.001**
**Arterial function and structure**				
Carotid-femoral PWV (cm/s)	753 ± 153	742 ± 148	−12 (−34, 11)	0.303
Brachial-ankle PWV (cm/s)^c^	1288 ± 158	1247 ± 163	**−41 (−63, −18)**	**0.001**
Carotid artery IMT (mm)	0.63 ± 0.13	0.61 ± 0.10	−0.01 (−0.02, 0.01)	0.519
Flow-mediated dilation (%)^bc^	4.3 ± 2.9	5.4 ± 3.5	**1.0 (0.1, 2.0)**	**0.030**
**Body weight and body composition**				
Body weight (kg)	91.7 ± 19.6	89.2 ± 18.8	−2.5 (−3.1, −1.9)	**< 0.001**
BMI (kg/m^2^)	34.4 ± 7.2	33.4 ± 6.9	−0.9 (−1.2, −0.7)	**< 0.001**
Waist circumference (cm)	109 ± 15	107 ± 15	−2 (−4, −1)	**0.004**
Body fat percentage (%)^c^	39 ± 8	39 ± 8	−0.3 (−0.6, −0.0)	**0.041**
Fat mass (kg)	36.6 ± 12.8	35.2 ± 12.7	−1.4 (−1.8, −1.0)	**< 0.001**
Muscle mass (kg)^c^	52.4 ± 10.0	51.4 ± 9.6	−1.0 (−1.5, −0.6)	**< 0.001**
**Blood pressure and heart rate**				
Systolic BP (mmHg)^c^	121 ± 11	117 ± 10	−5 (−7, −3)	**< 0.001**
Diastolic BP (mmHg)	70 ± 8	67 ± 7	−3 (−5, −2)	**< 0.001**
Mean BP (mmHg)	87 ± 8	84 ± 7	−4 (−5, −3)	**< 0.001**
Pulse pressure (mmHg)	51 ± 8	50 ± 8	−1 (−3, 0.2)	0.085
Heart rate at rest (beats/min)	63 ± 8	61 ± 8	−2 (−3, −1)	**0.008**
**Metabolic and inflammation profiles**				
Total cholesterol (mmol/l)	4.8 ± 1.1	4.5 ± 1.2	−0.3 (−0.5, −0.1)	**0.003**
HDL cholesterol (mmol/l)^c^	1.1 ± 0.4	1.0 ± 0.3	−0.05 (−0.11, 0.02)	0.144
Non-HDL cholesterol (mmol/l)	3.7 ± 1.1	3.5 ± 1.2	−0.25 (−0.45, −0.04)	**0.009**
LDL cholesterol (mmol/l)	3.1 ± 1.0	3.0 ± 1.1	−0.1 (−0.3, 0.1)	0.216
LDL/HDL cholesterol	3.1 ± 1.4	3.3 ± 1.9	0.2 (−0.2, 0.6)	0.415
Blood triglycerides (mmol/l)^c^	1.3 ± 0.6	1.1 ± 0.5	−0.3 (−0.4, −0.1)	**< 0.001**
Blood glucose (mmol/l)^c^	5.4 ± 1.0	5.3 ± 1.0	−0.2 (−0.3, 0.0)	0.055
Serum insulin (µU/ml)^ac^	17.7 ± 15.4	12.9 ± 10.0	−4.8 (−7.5, −2.0)	**0.001**
HOMA-IR^ac^	4.5 ± 3.4	3.3 ± 2.9	−0.8 (−1.3, −0.5)	**< 0.001**
C-reactive protein (mg/l)^ac^	4.3 ± 4.2	3.7 ± 3.8	−0.6 (−1.71, 0.5)	0.303
IP-10 (pg/ml)^ac^	44.9 ± 44.4	39.2 ± 35.5	−5.7 (−10.6, −0.8)	**0.023**
**Framingham risk score**				
Lipid model (%)^c^	8.4 ± 7.3	7.0 ± 4.7	−1.4 (−2.5, −0.3)	**0.012**
BMI model (%)^c^	10.2 ± 8.8	9.1 ± 7.6	−1.1 (−1.7, −0.5)	**< 0.001**

^a^Sample size is 41. ^b^ Sample size is 40. ^c^ Non-normally distributed variables; comparisons were analyzed using the Wilcoxon signed-rank tests. All other variables were analyzed using paired-sample t tests. Abbreviations: PWV, pulse wave velocity; IMT, intima media thickness; BMI, body mass index; BP, blood pressure; MAP, mean arterial pressure; HDL, high-density lipoprotein; LDL, low-density lipoprotein; HOMA-IR, homeostatic model assessment of insulin resistance; IP-10, interferon gamma-induced protein 10.

**Fig 3:**
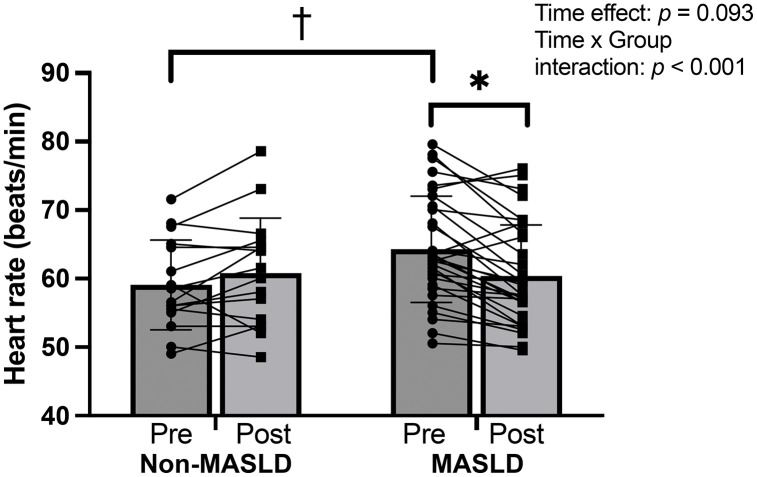
Different responses of heart rate after intervention in the non-MASLD and MASLD groups. Data were means ± SD. Comparisons were analyzed using a two-way ANOVA with one within-subject factor and one between-subject factor, followed by Bonferroni-adjusted post hoc comparisons. * *p* < 0.05 vs. pre in the same group. † p < 0.05 vs. non-MASLD group at the same time point.

With the exception of pulse pressure, most blood pressure measures and heart rate were significantly reduced following the intervention (Table 2). The reduction in heart rate was more pronounced in individuals with MASLD compared with those without (Two-way mixed ANOVA, *F*_*MASLD-time*_(1, 46) = 19.6, *p* < 0.001, partial *η²* = 0.30). Individuals with MASLD also had significantly higher baseline heart rates compared with those without MASLD (Fig 3). The blood concentrations of total cholesterol, triglycerides, and non-HDL cholesterol decreased significantly, whereas HDL cholesterol and LDL cholesterol concentrations remained unchanged (Table 2). While fasting glucose levels remained unchanged, insulin concentrations and insulin resistance index (HOMA-IR) decreased significantly (both *p* < 0.001) following the intervention (Table 2). For the inflammatory profile, serum IP-10 concentrations decreased significantly (Wilcoxon signed-rank test, *p* = 0.023, 95% CI: [−10.6, −0.8] pg/ml), while serum CRP concentrations did not reach statistical significance (Table 2). Individuals with and without MASLD showed similar responses to these measures following the intervention (S3 Table). Framingham Risk Scores for estimating risks decreased following the intervention in both the lipid-based model and the BMI-based model (Table 2). Individuals with MASLD and those without showed a similar response to the intervention in both models (S3 Table).

**Fig 4:**
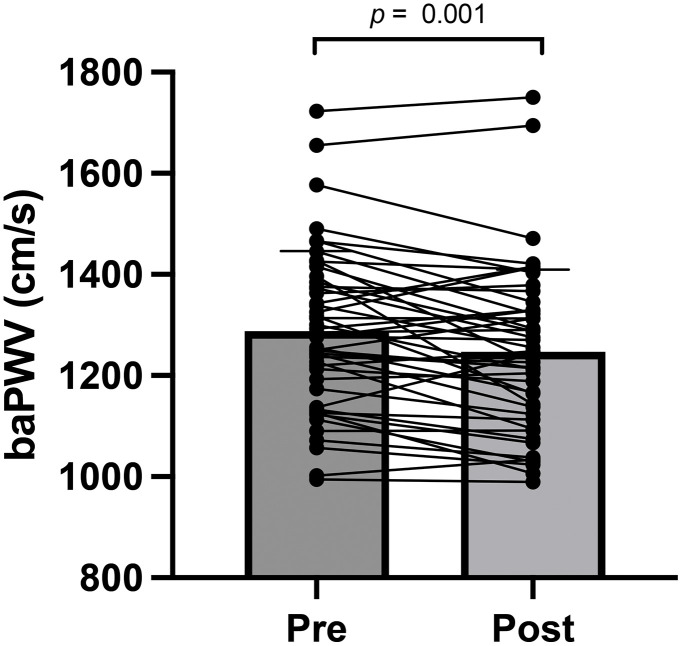
Arterial stiffness as assessed by brachial-ankle pulse wave velocity (baPWV) before and after the dietary interventions. Data were means ± SD. The comparison was analyzed using a non-parametric t test from paired samples (Wilcoxon signed-rank test).

**Fig 5:**
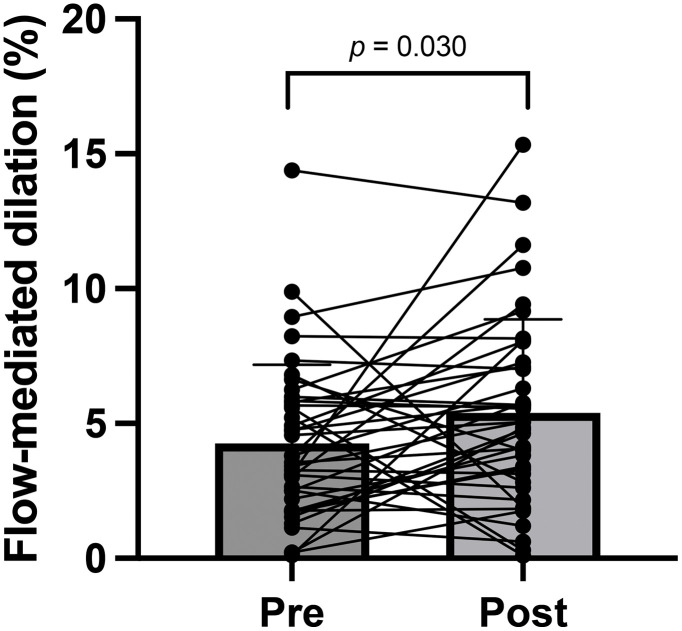
Endothelium-dependent vasodilation as assessed by brachial artery flow-mediated dilation (FMD) before and after the dietary interventions. Data were means ± SD. The comparison was analyzed using a non-parametric t-test for paired samples (Wilcoxon signed-rank test).

Brachial–ankle PWV decreased by an average of 41 cm/s (Wilcoxon signed-rank test, *p* = 0.001, 95% CI: [−63, −18] cm/s) (Table 2 and Fig 4), corresponding to a relative reduction of 3% (95% CI [−1, −5] %). Both diet groups (S2 Table) and MASLD status groups (S2 and S3 Tables) showed similar responses to the intervention with respect to brachial–ankle PWV. No significant changes were observed in carotid-femoral PWV and carotid IMT following the intervention (Table 2), and the subgroup analyses showed similar results (S2 and S3 Tables). Brachial FMD significantly increased (Wilcoxon signed-rank test, *p* = 0.030, 95% CI: [0.1, 2.0] %) (Table 2 and Fig 5). Both diet groups showed similar responses to the intervention for FMD (S2 Table). In participants in MASLD, subgroup analyses yielded comparable findings to those observed in the overall cohort. However, the increase in FMD did not reach statistical significance (n = 26, *p* = 0.067, 95%CI: [−0.1, 3.2] %). Detailed results are provided in the Supporting Information (S4 Table).

**Fig 6:**
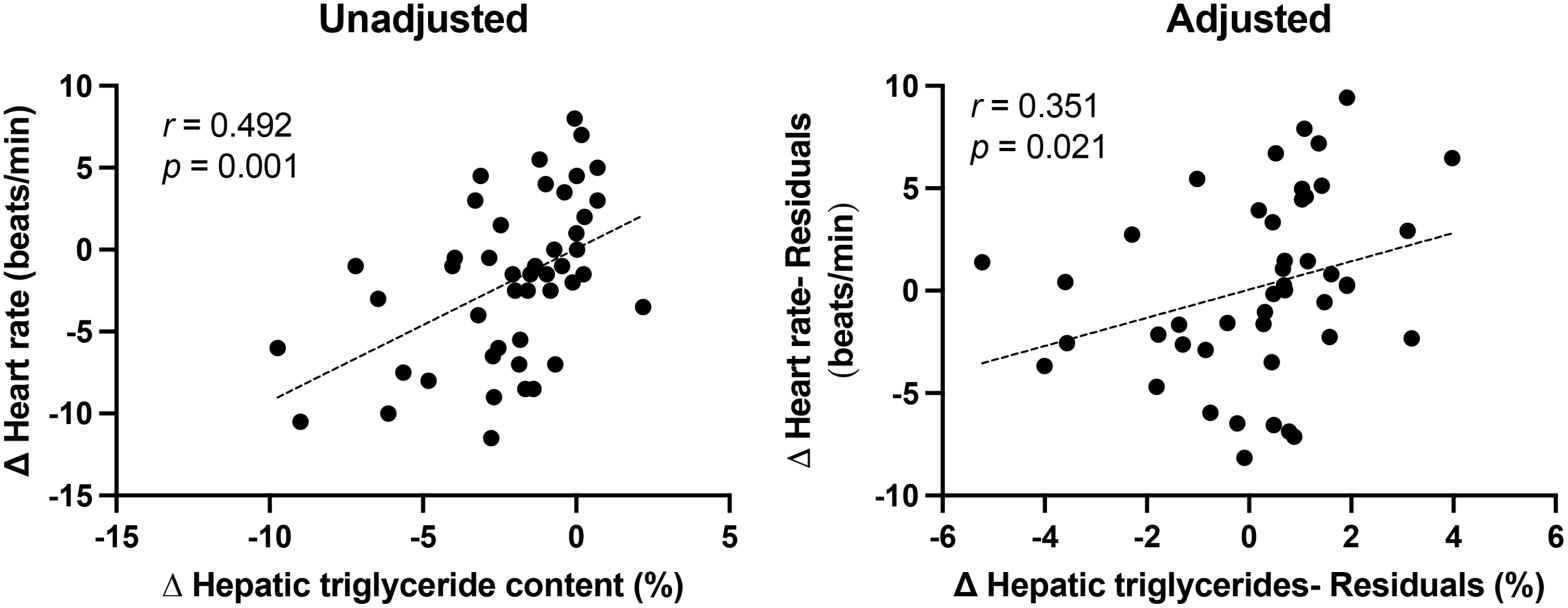
Correlations between changes in hepatic triglyceride content and heart rate. (A) Spearman correlation without adjusted factors. (B) Partial correlation after adjusting for age and body weight change. Residuals (adjusted for age and body weight change) were used to visualize the partial correlation results between the variables.

In the current study, the changes were calculated by post-intervention values minus the pre-intervention values. The associations between hepatic triglyceride reduction and heart rate change remained significant before (Spearman correlation, *r* = 0.492, *p* = 0.001) and after controlling for age and body weight change (Partial correlation, *r* = 0.35, *p* = 0.021) (Fig 6). Changes in hepatic triglycerides were not significantly associated with changes in brachial-ankle PWV (Spearman correlation, *r* = 0.014, *p* = 0.926) and FMD (Spearman correlation, *r* = 0.157, *p* = 0.340). In the correlation analysis between changes FMD and hepatic triglycerides, one outlier was excluded. The association remained insignificant when the outlier was retained. Among participants with MASLD, the absence of significant associations between changes in hepatic triglycerides and changes in brachial-ankle PWV or FMD was consistent with the findings in the overall cohort. However, the association between changes in hepatic triglycerides and heart rate was no longer statistically significant (S5 Table).

## Discussion

This study investigated changes in hepatic triglyceride content, cardiometabolic risk factors, and subclinical cardiovascular profiles in individuals with or at high risk of MASLD following a two-week dietary intervention. Participants experienced significant reductions in hepatic triglyceride content along with the reductions in body weight, cardiometabolic risk factors, arterial stiffness and estimated 10-year cardiovascular disease risk, and improvements in endothelial function. Alterations in heart rate at rest were positively associated with hepatic triglyceride change, even after adjustment for age and body weight change. The changes in vascular measures were not correlated with the changes in hepatic triglycerides.

In the current study, a two-week weight loss intervention induced a reduction of hepatic triglycerides, which was accompanied by improvements in brachial-ankle PWV and FMD—both are established predictors of future cardiovascular risk. We observed 1% improvement in FMD, aligning with prior meta-analyses linking such changes to a ~ 9% reduction in future cardiovascular risk [16]. This finding aligns with the limited interventional evidence examining vascular responses in MASLD. One recent long-term nutrition supplement study showed that taking high-dose coenzyme Q10 for six months induced concurrent improvements in FMD along with reductions in hepatic triglycerides [17]. This finding extends previous observational evidence showing inverse associations between hepatic fat and endothelial function [18], indicating that regression of hepatic triglycerides may directly benefit vascular health in MASLD. Although a significant correlation between changes in hepatic triglycerides and FMD was not observed in the present study, the relatively short intervention duration and modest magnitude of FMD improvement may have limited the ability to detect such an association. Although the improvement in FMD did not reach statistical significance in the MASLD subgroup analysis (S4 Table), the direction and magnitude of change were similar to those observed in the overall cohort. Given the limited sample size (n = 26) and borderline statistical significance (*p* = 0.067), larger studies are warranted to confirm this finding. Collectively, these findings suggest that the short-term weight-loss intervention commonly applied in MASLD management may also be promising to improve vascular function. However, whether the observed vascular improvement is directly attributable to the reduction of hepatic triglycerides remains uncertain. Further molecular-level studies are warranted to delineate the mechanistic pathways underlying potential liver–vascular interaction.

The mechanisms linking MASLD to vascular dysfunction are not fully understood. One prevailing theory posits that dyslipidemia and inflammation are key mediators of this relation [19]. Accumulation of hepatic fat promotes the overproduction and remodeling of very low-density lipoprotein (VLDL) [20,21], leading to an increase in small, dense low-density lipoprotein (sdLDL) particles—among the most atherogenic lipoproteins associated with cardiovascular disease risk [22,23]. This results in a highly atherogenic lipid profile that may contribute to endothelial injury. Consistent with this, MASLD has been independently associated with elevated sdLDL concentrations [24]. Another proposed mechanism revolves around elevated inflammation. The liver contains one of the largest populations of immune cells in the body—Kupffer cells—which secrete proinflammatory cytokines in response to excess fatty acid influx [25]. Excess hepatic lipid accumulation activates Kupffer cells through lipotoxic and Toll-like receptor 4 (TLR4)–mediated pathways, initiating intrahepatic inflammation that propagates systemically through cytokine release [25]. This immune activation contributes to elevated circulating inflammatory markers, which can impair endothelial nitric oxide signaling and promote vascular dysfunction [26]. The overall trend toward reduced inflammation in the present study supports inflammation as a potential contributor to improved vascular function.

In the current study, people with MASLD had a higher heart rate at baseline, and a significant reduction in heart rate at rest was observed only among individuals with MASLD. Elevations in heart rate at rest among individuals with MASLD have been previously reported [27]. The mechanisms underlying this association remain unclear but may involve elevated sympathetic and/or reduced parasympathetic activities commonly observed in metabolic disorders [28]. Another proposed mechanism relates to the alterations in hepatic hemodynamics. Hepatic triglyceride accumulation may enlarge hepatocytes and compress hepatic sinusoids, potentially impairing venous outflow and reducing cardiac preload. This reduction in preload could theoretically elicit compensatory sympathetic activation, contributing to an elevated heart rate at rest [29]. According to this theory, the association between reductions in hepatic triglyceride content and heart rate at rest observed in the present study may reflect improvements in hepatic hemodynamics and autonomic regulation following hepatic triglyceride reduction. Conversely, experimental studies in animal models suggest the autonomic nervous system has a direct role in hepatic triglyceride synthesis [30] and inflammation [31]. However, evidence for these interactions between hepatic triglycerides and the autonomic nervous system in humans remains limited. These hypothesis-generating observations warrant further mechanistic exploration to determine whether improvements in liver health can modulate autonomic tone and, in turn, confer cardiovascular benefits in humans.

To our knowledge, this is the first dietary intervention study to investigate clinically significant vascular measures in individuals with MASLD and to directly correlate their alterations using MRI-based quantification. Approximately 97% of participants with MASLD exhibited a reduction in hepatic triglyceride content. This favorable response may reflect the short duration of the intervention, pre-prepared meals, and the customized dietary content according to participants’ preferences. However, this study also has several limitations. First, the sample was small and predominantly female, which may reduce the statistical power and generalizability of the findings, particularly given the prevalence of MASLD being higher in men [32]. In addition, the sample size calculation was based on the expected change in hepatic triglyceride content, the primary mechanistic target of the intervention. Therefore, the study may have been underpowered to detect modest changes in some secondary outcomes and associations of interest. Second, the short-term intervention may not have been sufficient to affect central stiffness, as ~6–8% weight loss is typically required to improve central arterial stiffness, with BMI or body weight change identified as key predictors in long-term trials [33] and a meta-analysis [34]. Only ~3% weight loss was achieved in the present study. Third, the study’s correlational analyses and design restrict causal inference. Future randomized controlled trials with larger and more diverse populations are needed to confirm these findings and clarify the underlying mechanisms.

## Conclusions

A two-week dietary intervention (either a low-calorie or low-carbohydrate diet) was successful in reducing hepatic triglyceride content among individuals with or at high risk of MASLD. The reductions in hepatic triglycerides were accompanied by the corresponding improvements in arterial stiffness, endothelial function, and cardiovascular disease risk. The independent association between changes in hepatic triglycerides and heart rate suggests a connection between MASLD and autonomic regulation.

## Supporting information

S1 File Supporting Information_TREND Statement Checklist. (PDF)

S1 Table Participant characteristics grouped by diet assignment. (DOCX)

S2 Table Changes in hepatic, metabolic, and cardiovascular outcomes from pre- to post-intervention grouped by diet assignment. (DOCX)

S3 Table Changes in hepatic, metabolic, and cardiovascular outcomes from pre to post dietary interventions grouped by MASLD status. (DOCX)

S4 Table Changes in hepatic, metabolic, and cardiovascular outcomes from pre- to post-intervention in people with MASLD. (DOCX)

S5 Table Pearson correlation between changes in hepatic triglycerides and heart rate, brachial ankle pulse wave velocity and flow-mediated dilation in people with MASLD. (DOCX)
