# Supplementary material for: The impact of short-term reduction in hepatic triglycerides on vascular health in adults with or at risk of metabolic dysfunction-associated steatotic liver disease
Source: PLoS One. 2026 Aug 13;21(8):e0345338. doi: 10.1371/journal.pone.0345338 (PMC13472412; doi:10.1371/journal.pone.0345338)
Supplement: Supplementary file 2 [file pone.0345338.s002.docx]

**S1 Table. Participant characteristics grouped by diet assignment.**

|  | **Low calorie (n=23)** | **Low carbohydrate (n=24)** | ***p* value** |
| --- | --- | --- | --- |
| Age (years) | 52 ± 7 | 55 ± 9 | 0.233 |
| Female/Male (n, %) | 19/4 (83/17) | 19/5 (79/21) | 0.740 |
| Race (n, %) |  |  | 0.549 |
| White | 13 (57) | 16 (67) |  |
| Blacks | 3 (13) | 4 (17) |  |
| Asian | 1 (4) | 2 (8) |  |
| Other | 6 (26) | 2 (8) |  |
| Body weight (kg) | 90.8 ± 24.5 | 92.5 ± 14.0 | 0.775 |
| Height (cm) | 163.5 ± 8.7 | 163.3 ± 6.6 | 0.947 |
| BMI (kg/m^2^) | 33.9 ± 8.3 | 34.8 ± 6.1 | 0.648 |
| Waist circumference (cm) | 107.1 ± 18.0 | 111.6 ± 10.8 | 0.311 |
| Body fat percentage (%) | 39.4 ± 8.7 | 38.5 ± 7.7 | 0.350 |
| Systolic BP (mmHg) | 116.6 ± 9.0 | 126.1 ± 11.6 | **0.002** |
| Diastolic BP (mmHg) | 67.2 ± 7.3 | 73.4 ± 6.5 | **0.002** |
| Total cholesterol (mmol/l) | 4.9 ± 1.2 | 4.8 ± 1.1 | 0.390 |
| HDL cholesterol (mmol/l)^b^ | 1.1 ± 0.4 | 1.0 ± 0.4 | 0.437 |
| LDL cholesterol (mmol/l)^b^ | 3.2 ± 1.1 | 3.1 ± 0.9 | 0.710 |
| LDL/HDL cholesterol^b^ | 3.1 ± 1.5 | 3.2 ± 1.3 | 0.225 |
| Blood triglycerides (mmol/l)^b^ | 1.2 ± 0.4 | 1.4 ± 0.7 | 0.632 |
| Blood glucose (mmol/l)^b^ | 5.2 ± 0.5 | 5.7 ± 1.3 | 0.176 |
| Plasma insulin (µU/ml)^ab^ | 17.0 ± 11.2 | 18.5 ± 19.7 | 0.638 |
| HOMA-IR^ab^ | 4.0 ± 3.0 | 4.8 ± 6.0 | 0.937 |
| C-reactive protein (mg/l)^ab^ | 4.6 ± 3.4 | 3.9 ± 5.0 | 0.418 |
| Hepatic triglycerides (%)^b^ | 12.0 ± 9.8 | 13.1 ± 11.2 | 1.000 |
| MASLD (n, %) | 16 (61) | 15 (63) | 0.609 |

Data are displayed as n (%) or median (interquartile range). ^a^ Sample size: Sample size: low-calorie group = 19, and low-carbohydrate group = 22. ^b^ Non-normally distributed variable; comparisons were performed using the Mann-Whitney U test. All other continuous variables were analyzed using independent-sample t tests. Categorical variables were compared using the chi-square test. Abbreviations: BP, blood pressure; HDL, high-density lipoprotein; LDL, low-density lipoprotein; HOMA-IR, homeostatic model assessment of insulin resistance; IP-10, interferon gamma-induced protein 10; MASLD, metabolic dysfunction-associated steatotic liver disease
