# Supplementary material for: The impact of short-term reduction in hepatic triglycerides on vascular health in adults with or at risk of metabolic dysfunction-associated steatotic liver disease
Source: PLoS One. 2026 Aug 13;21(8):e0345338. doi: 10.1371/journal.pone.0345338 (PMC13472412; doi:10.1371/journal.pone.0345338)
Supplement: Supplementary file 3 [file pone.0345338.s003.docx]

**S2 Table.** **Changes in hepatic, metabolic, and cardiovascular outcomes from pre- to post-intervention grouped by diet assignment.**

|  | **Low Calorie Diet**  **(n=23)** | | |  | **Low Carbohydrate Diet**  **(n=24)** | |  | **Time main effect** |  | **Time * group interaction** |
| --- | --- | --- | --- | --- | --- | --- | --- | --- | --- | --- |
|  | **Pre** | | **Post** |  | **Pre** | **Post** |  | *p* value |  | *p* value |
| Hepatic triglycerides (%) | 12.0 ± 9.8 | | 9.4 ± 7.6* |  | 13.1 ± 11.2 | 11.4 ± 9.9* |  | **< 0.001** |  | 0.255 |
| **Body weight and body composition** | | | | | | | | | |  |
| Body weight (kg) | 90.8 ± 24.5 | 88.7 ± 23.3* | |  | 92.5 ± 14.0 | 89.7 ± 13.6* |  | **< 0.001** |  | 0.242 |
| BMI (kg/m^2^) | 33.9 ± 8.3 | 33.1 ± 8.0* | |  | 34.8 ± 6.1 | 33.8 ± 5.8* |  | **< 0.001** |  | 0.182 |
| Waist circumference (cm) | 105.4 ± 16.4 | 103.7 ± 16.7 | |  | 112.2 ± 10.6 | 109.7 ± 11.9* |  | **0.005** |  | 0.572 |
| Hip circumference (cm) | 114.3 ± 13.8 | 114.2 ± 12.7 | |  | 117.6 ± 11.1 | 117.0 ± 11.3 |  | 0.523 |  | 0.652 |
| Waist-to-hip ratio | 0.92 ± 0.08 | 0.91 ± 0.09* | |  | 0.96 ± 0.06 | 0.94 ± 0.07* |  | **0.004** |  | 0.884 |
| Body fat (%) | 39.4 ± 8.7 | 39.0 ± 9.0 | |  | 38.5 ± 7.7 | 38.3 ± 7.9 |  | **0.040** |  | 0.562 |
| Fat mass (kg) | 36.9 ± 14.8 | 35.4 ± 14.8* | |  | 36.2 ± 11.0 | 34.9 ± 10.6* |  | **< 0.001** |  | 0.695 |
| Muscle mass (kg) | 51.2 ± 12.4 | 50.6 ± 11.7 | |  | 53.5 ± 7.1 | 52.1 ± 7.2* |  | **< 0.001** |  | 0.100 |
| Visceral fat score (AU) | 12.0 ± 4.7 | 11.5 ± 4.3* | |  | 12.1 ± 2.9 | 11.8 ± 2.7* |  | **< 0.001** |  | 0.424 |
| **Blood pressures and heart rate** | | | | | | | | | |  |
| Systolic BP (mmHg) | 117 ± 9 | 114 ± 9* | |  | 126 ± 12† | 120 ± 11*† |  | **< 0.001** |  | 0.095 |
| Diastolic BP (mmHg) | 67 ± 7 | 64 ± 7* | |  | 73 ± 7† | 69 ± 5*† |  | **< 0.001** |  | 0.355 |
| Mean BP (mmHg) | 84 ± 7 | 81 ± 7* | |  | 91 ± 7† | 86 ± 6*† |  | **< 0.001** |  | 0.165 |
| Pulse pressure (mmHg) | 49 ± 6 | 49 ± 6 | |  | 53 ± 9 | 50 ± 10* |  | 0.087 |  | 0.168 |
| Heart rate (beats/min) | 64 ± 8 | 61 ± 8* | |  | 62 ± 7 | 61 ± 7 |  | **0.007** |  | 0.144 |
| **Metabolic and inflammation profiles** | | | | | | | | | |  |
| Total cholesterol (mmol/l) | 4.9 ± 1.2 | 4.6 ± 1.3 | |  | 4.8 ± 1.1 | 4.4 ± 1.1* |  | **0.004** |  | 0.706 |
| HDL cholesterol (mmol/l) | 1.1 ± 0.4 | 1.0 ± 0.4* | |  | 0.9 ± 0.4 | 1.1 ± 0.3 |  | 0.119 |  | **0.035** |
| Non-HDL (mmol/l) | 3.7 ± 1.1 | 3.6 ± 1.3 | |  | 3.7 ± 1.1 | 3.4 ± 1.1* |  | **0.020** |  | 0.342 |
| LDL cholesterol (mmol/l) | 3.2 ± 1.1 | 3.1 ± 1.2 | |  | 3.1 ± 0.9 | 2.9 ± 1.0 |  | 0.224 |  | 0.614 |
| LDL/HDL cholesterol | 3.1 ± 1.5 | 3.6 ± 2.2 | |  | 3.2 ± 1.3 | 3.0 ± 1.6 |  | 0.381 |  | 0.066 |
| Blood triglycerides (mmol/l) | 1.2 ± 0.4 | 1.1 ± 0.5 | |  | 1.4 ± 0.7 | 1.1 ± 0.4* |  | **< 0.001** |  | 0.058 |
| Blood glucose (mmol/l) | 5.2 ± 0.5 | 5.1 ± 0.5 | |  | 5.7 ± 1.3 | 5.4 ± 1.3* |  | 0.056 |  | 0.160 |
| Serum insulin (µU/ml)^a^ | 17.0 ± 11.2 | 14.9 ± 10.1 | |  | 18.5 ± 19.7 | 14.9 ± 10.7* |  | **< 0.001** |  | **0.030** |
| HOMA IR (AU)^a^ | 4.0 ± 3.0 | 3.5 ± 2.6 | |  | 4.8 ± 6.0 | 2.5 ± 2.6* |  | **0.002** |  | 0.051 |
| IP-10 (pg/ml)^a^ | 51.4 ± 56.8 | 47.1 ± 45.5 | |  | 34.6 ± 14.3 | 29.5 ± 12.3* |  | **0.021** |  | 0.519 |
| C-reactive protein (mg/l)^a^ | 4.6 ± 3.4 | 4.0 ± 4.2 | |  | 3.9 ± 5.0 | 3.3 ± 3.3 |  | 0.331 |  | 0.990 |
| **Arterial function and structure** | | | | | | | | | |  |
| Carotid-femoral PWV (cm/s) | 713 ± 106 | 717 ± 96 | |  | 789 ± 181 | 764 ± 183 |  | 0.329 |  | 0.184 |
| Brachial-ankle PWV (cm/s) | 1238 ± 141 | 1195 ± 128* | |  | 1336 ± 162† | 1298 ± 179*† |  | **< 0.001** |  | 0.859 |
| Carotid artery IMT (mm) | 0.60 ± 0.11 | 0.60 ± 0.10 | |  | 0.65 ± 0.14 | 0.62 ± 0.11 |  | 0.294 |  | 0.144 |
| Flow-mediated dilation (%)^b^ | 4.8 ± 3.7 | 5.0 ± 3.7 | |  | 3.8 ± 1.9 | 5.8 ± 3.3* |  | 0.051 |  | 0.096 |
| **Framingham risk score** | | | | | | | | | |  |
| Lipid model | 5.8 ± 2.6 | 5.5 ± 2.5 | |  | 11.0 ± 9.3† | 8.4 ± 5.9*† |  | **0.011** |  | **0.037** |
| BMI model | 7.2 ± 3.9 | 6.6 ± 3.4 | |  | 13.1 ± 11.0† | 11.4 ± 9.6*† |  | **< 0.001** |  | 0.081 |

Data are means ± SD. ^a^ Sample size: low-calorie group = 19, and low-carbohydrate group = 22. ^b^ Sample size: low-calorie group = 20, and low-carbohydrate group = 20. Abbreviations: BMI, body mass index; BP, blood pressure; HDL, high-density lipoprotein; LDL, low-density lipoprotein; HOMA-IR, homeostatic model assessment of insulin resistance; IP-10, interferon gamma-induced protein 10; PWV, pulse wave velocity; IMT, intima-media thickness. Two-way mixed ANOVA (one within and one between-subject factor) with Bonferroni correction for post-hoc test was performed. * *p*<0.05 for within-group comparison between baseline and post-intervention; † *p*<0.05 for between-group comparison at the same time point.
