# Supplementary material for: The impact of short-term reduction in hepatic triglycerides on vascular health in adults with or at risk of metabolic dysfunction-associated steatotic liver disease
Source: PLoS One. 2026 Aug 13;21(8):e0345338. doi: 10.1371/journal.pone.0345338 (PMC13472412; doi:10.1371/journal.pone.0345338)
Supplement: Supplementary file 4 [file pone.0345338.s004.docx]

**S3 Table.** **Changes in hepatic, metabolic, and cardiovascular outcomes from pre to post dietary interventions grouped by MASLD status.**

|  | **Non-MASLD**  **(n = 16)** | |  | **MASLD**  **(n = 31)** | |  | **Time main effect** |  | **Time * group interaction** | |
| --- | --- | --- | --- | --- | --- | --- | --- | --- | --- | --- |
|  | **Pre** | **Post** |  | **Pre** | **Post** |  | ***p* value** |  | ***p* value** | |
| Hepatic triglycerides (%) | 2.7 ± 1.2 | 2.4 ± 1.2 |  | 17.9 ± 9.3† | 14.7 ± 8.1*† |  | **< 0.001** |  | **< 0.001** | |
| **Body weight and body composition** | | | | | | | | | |  |
| Body weight (kg) | 80.9 ± 17.5 | 79.0 ± 16.9* |  | 97.3 ± 18.5† | 94.4 ± 17.8*† |  | **< 0.001** |  | 0.141 | |
| BMI (kg/m^2^) | 30.5 ± 6.8 | 29.8 ± 6.6* |  | 36.3 ± 6.6† | 35.3 ± 6.4*† |  | **< 0.001** |  | 0.135 | |
| Waist circumference (cm) | 100.4 ± 13.9 | 97.7 ± 14.4* |  | 113.6 ± 11.9† | 111.8 ± 12.2† |  | **0.004** |  | 0.485 | |
| Hip circumference (cm) | 110.4 ± 14.5 | 110.3 ± 13.8 |  | 119.1 ± 10.2 | 118.5 ± 9.9 |  | 0.621 |  | 0.659 | |
| Waist to hip ratio | 0.91 ± 0.07 | 0.89 ± 0.09* |  | 0.95 ± 0.07 | 0.94 ± 0.07† |  | **0.001** |  | 0.181 | |
| Body fat (%) | 35.2 ± 9.8 | 34.9 ± 10.1 |  | 40.9 ± 6.4† | 40.6 ± 6.7† |  | 0.055 |  | 0.967 | |
| Fat mass (kg) | 29.5 ± 12.8 | 28.2 ± 12.9* |  | 40.2 ± 11.4† | 38.7 ± 11.1*† |  | **< 0.001** |  | 0.579 | |
| Muscle mass (kg) | 48.8 ± 8.4 | 48.4 ± 7.6 |  | 54.2 ± 10.4 | 52.9 ± 10.2* |  | **0.001** |  | 0.091 | |
| Visceral fat score (AU) | 10.3 ± 3.5 | 10.1 ± 3.3 |  | 13.0 ± 3.7† | 12.4 ± 3.5*† |  | **< 0.001** |  | **0.023** | |
| **Blood pressure and heart rate** | | | | | | | | | |  |
| Systolic BP (mmHg) | 118 ± 12 | 113 ± 7* |  | 123 ± 11† | 118 ± 11* |  | **< 0.001** |  | 0.782 | |
| Diastolic BP (mmHg) | 69 ± 8 | 67 ± 7 |  | 71 ± 7 | 67 ± 6* |  | **< 0.001** |  | 0.087 | |
| Mean BP (mmHg) | 85 ± 9 | 83 ± 7* |  | 88 ± 8 | 84 ± 7* |  | **< 0.001** |  | 0.236 | |
| Pulse pressure (mmHg) | 49 ± 8 | 46 ± 6 |  | 52 ± 8 | 53 ± 10† |  | 0.052 |  | 0.308 | |
| Heart rate (beats/min) | 59 ± 7 | 61 ± 8 |  | 64 ± 8† | 61 ± 7* |  | 0.093 |  | **< 0.001** | |
| **Metabolic and inflammation profiles** | | | | | | | | | |  |
| Total cholesterol (mmol/l) | 5.0 ± 1.2 | 4.8 ± 1.3 |  | 4.7 ± 1.1 | 4.3 ± 1.1* |  | **0.011** |  | 0.347 | |
| HDL cholesterol (mmol/l) | 1.1 ± 0.4 | 1.1 ± 0.3 |  | 1.1 ± 0.4 | 1.0 ± 0.3* |  | 0.345 |  | 0.147 | |
| Non-HDL (mmol/l) | 3.9 ± 1.3 | 3.7 ± 1.4 |  | 3.6 ± 1.0 | 3.3 ± 1.1* |  | **0.037** |  | 0.670 | |
| LDL cholesterol (mmol/l) | 3.4 ± 1.2 | 3.3 ± 1.3 |  | 2.9 ± 0.9 | 2.8 ± 1.0 |  | 0.258 |  | 0.923 | |
| LDL/HDL cholesterol | 3.5 ± 1.9 | 3.5 ± 2.3 |  | 3.0 ± 1.0 | 3.2 ± 1.7 |  | 0.578 |  | 0.505 | |
| Blood triglyceride (mmol/l) | 1.1 ± 0.5 | 0.9 ± 0.3 |  | 1.5 ± 0.6† | 1.2 ± 0.5*† |  | **0.001** |  | 0.332 | |
| Blood glucose (mmol/l) | 5.2 ± 0.9 | 5.0 ± 0.7 |  | 5.6 ± 1.0 | 5.4 ± 1.0 |  | 0.059 |  | 0.767 | |
| Serum insulin (µU/mL)^a^ | 7.5 ± 2.9 | 6.2 ± 2.6 |  | 23.1 ± 16.7† | 16.5 ± 10.7*† |  | **0.007** |  | 0.062 | |
| HOMA-IR (AU)^a^ | 1.8 ± 0.9 | 1.4 ± 0.8 |  | 5.7 ± 5.1† | 3.9 ± 2.8*† |  | **0.017** |  | 0.106 | |
| IP-10 (pg/ml)^a^ | 54.8 ± 69.1 | 44.8 ± 51.8* |  | 39.6 ± 22.7 | 36.1 ± 23.2 |  | **0.011** |  | 0.195 | |
| C-reactive protein (mg/l)^a^ | 3.9 ± 5.8 | 2.9 ± 2.7 |  | 4.5 ± 3.0 | 4.1 ± 4.3 |  | 0.257 |  | 0.586 | |
| **Framingham risk score** | | | | | | | | | |  |
| Lipid model | 7.7 ± 6.6 | 6.2 ± 3.9 |  | 8.8 ± 7.7 | 7.4 ± 5.1* |  | **0.018** |  | 0.987 | |
| BMI model | 8.6 ± 7.5 | 7.6 ± 6.7 |  | 11.1 ± 9.3 | 9.9 ± 8.0* |  | **0.001** |  | 0.692 | |
| **Arterial function and structure** | | | | | | | | | |  |
| Carotid-femoral PWV (cm/s) | 728 ± 110 | 732 ± 110 |  | 767 ± 173 | 747 ± 167 |  | 0.493 |  | 0.286 | |
| Brachial-ankle PWV (cm/s) | 1252 ± 142 | 1227 ± 132 |  | 1306 ± 165 | 1258 ± 178* |  | **0.003** |  | 0.323 | |
| Carotid artery IMT (mm) | 0.59 ± 0.07 | 0.59 ± 0.05 |  | 0.64 ± 0.15 | 0.63 ± 0.12 |  | 0.423 |  | 0.518 | |
| Flow-mediated dilation (%)^b^ | 4.9 ± 3.5 | 5.3 ± 3.3 |  | 3.9 ± 2.5 | 5.5 ± 3.6* |  | 0.123 |  | 0.332 | |

Data are displayed as means ± SD. ^a^ Sample size: non-MASLD group = 14, and MASLD group = 27. ^b^ Sample size: non-MASLD group = 14, and MASLD group = 26. Abbreviations: BMI, body mass index; BP, blood pressure; HDL, high-density lipoprotein; LDL, low-density lipoprotein; HOMA-IR, homeostatic model assessment of insulin resistance; IP-10, interferon gamma-induced protein 10; PWV, pulse wave velocity; IMT, intima-media thickness. Two-way mixed ANOVA (one within and one between-subject factor)with Bonferroni correction for post hoc test was performed. * *p*<0.05 for within-group comparison between baseline and post-intervention; † *p*<0.05 for between-group comparison at the same time point.
