# Supplementary material for: The impact of short-term reduction in hepatic triglycerides on vascular health in adults with or at risk of metabolic dysfunction-associated steatotic liver disease
Source: PLoS One. 2026 Aug 13;21(8):e0345338. doi: 10.1371/journal.pone.0345338 (PMC13472412; doi:10.1371/journal.pone.0345338)
Supplement: Supplementary file 5 [file pone.0345338.s005.docx]

**S4 Table. Changes in hepatic, metabolic, and cardiovascular outcomes from pre- to post-intervention in people with MASLD.**

| **Mean ± SD** | **Pre**  **(n = 31)** | **Post**  **(n = 31)** | **Mean difference (95% CI)** | ***p* value** |
| --- | --- | --- | --- | --- |
| **Hepatic triglycerides** (%)^c^ | 18.3 ± 9.4 | 14.9 ± 8.2 | -3.2 (-4.1, -2.2) | **< 0.001** |
| **Arterial function and structure** | | | | |
| Carotid-femoral PWV (cm/s) | 767 ± 173 | 747 ± 167 | -20 (-42, 1) | 0.060 |
| Brachial-ankle PWV (cm/s) | 1306 ± 165 | 1258 ± 178 | -48 (-77, -20) | **0.002** |
| Carotid IMT (mm)^c^ | 0.64 ± 0.15 | 0.63 ± 0.12 | -0.01 (-0.03, 0.02) | 0.400 |
| Flow-mediated dilation (%)^bc^ | 3.9 ± 2.5 | 5.5 ± 3.6 | 1.5 (-0.1, 3.2) | 0.067 |
| **Body weight and body composition** | | | | |
| Body weight (kg) | 97.3 ± 18.5 | 94.4 ± 17.8 | -2.8 (-3.7, -2.0) | **< 0.001** |
| BMI (kg/m^2^) | 36.3 ± 6.6 | 35.3 ± 6.4 | -1.1 (-1.4, -0.8) | **< 0.001** |
| Waist circumference (cm) | 114 ± 12 | 112 ± 12 | -2 (-3, -0.1) | **0.033** |
| Body fat percentage (%) | 41 ± 6 | 41 ± 7 | -0.3 (-0.7, 0.1) | 0.086 |
| Fat mass (kg) | 40.2 ± 11.4 | 38.7 ± 11.1 | -1.5 (-1.9, -1.0) | **< 0.001** |
| Muscle mass (kg) | 54.2 ± 10.4 | 52.9 ± 10.2 | -1.3 (-1.9, -0.7) | **< 0.001** |
| **Blood pressure and heart rate** | | | | |
| Systolic BP (mmHg) | 123 ± 11 | 118 ± 11 | -5 (-7, -3) | **< 0.001** |
| Diastolic BP (mmHg) | 71 ± 7 | 67 ± 6 | -4 (-6, -3) | **< 0.001** |
| Mean BP (mmHg) | 88 ± 8 | 84 ± 7 | -5 (-6, -3) | **< 0.001** |
| Pulse pressure (mmHg) | 52 ± 8 | 52 ± 9 | -1 (-3, 1) | 0.369 |
| Heart rate (beats/min) | 64 ± 8 | 61 ± 7 | -4 (-5, -2) | **< 0.001** |
| **Metabolic and inflammation profiles** | | | | |
| Total cholesterol (mmol/l) | 4.9 ± 1.1 | 4.3 ± 1.1 | -0.4 (-0.6, -0.1) | **0.005** |
| HDL cholesterol (mmol/l)^c^ | 1.1 ± 0.4 | 1.0 ± 0.3 | -0.10 (-0.19, -0.01) | **0.027** |
| Non-HDL cholesterol (mmol/l) | 3.6 ± 1.0 | 3.3 ± 1.1 | -0.3 (-0.5, -0.02) | **0.039** |
| LDL cholesterol (mmol/l)^c^ | 2.9 ± 0.9 | 2.8 ± 1.0 | -0.18 (-0.36, 0.03) | 0.082 |
| LDL/HDL cholesterol^c^ | 3.0 ± 1.0 | 3.2 ± 1.7 | 0.04 (-0.22, 0.56) | 0.604 |
| Blood triglycerides (mmol/l)^c^ | 1.5 ± 0.6 | 1.2 ± 0.5 | -0.31 (-0.43, -0.18) | **< 0.001** |
| Blood glucose (mmol/l)^c^ | 5.6 ± 1.0 | 5.4 ± 1.0 | -0.08 (-0.39, 0.11) | 0.409 |
| Serum insulin (µU/ml)^ac^ | 23.6 ± 16.6 | 17.1 ± 10.9 | -4.7 (-7.0, -2.4) | **< 0.001** |
| HOMA-IR index^ac^ | 5.8 ± 5.0 | 4.2 ± 3.2 | -1.2 (-1.8, -0.6) | **< 0.001** |
| C-reactive protein (mg/l)^ac^ | 4.9 ± 3.6 | 4.3 ± 4.3 | -0.8 (-1.4, 0.1) | 0.099 |
| IP-10 (pg/ml)^ac^ | 40.1 ± 22.4 | 36.0 ± 22.8 | -3.0 (-5.5, -0.6) | **0.006** |
| **Framingham risk score** | | | | |
| Lipid model (%)^c^ | 8.8 ± 7.7 | 7.4 ± 5.1 | -0.9 (-1.5, -0.3) | **0.011** |
| BMI model (%)^c^ | 11.1 ± 9.3 | 9.9 ± 8.0 | -0.9 (-1.6, -0.4) | **0.001** |

^a^ Sample size is 27. ^b^ Sample size is 26. ^c^ Non-normally distributed variables; comparisons were analyzed using the Wilcoxon signed-rank tests. All other variables were analyzed using paired-sample t tests. Abbreviations: BMI, body mass index; BP, blood pressure; MAP, mean arterial pressure; HDL, high-density lipoprotein; LDL, low-density lipoprotein; HOMA-IR, homeostatic model assessment of insulin resistance; IP-10, interferon gamma-induced protein 10; PWV, pulse wave velocity; IMT, intima media thickness.
