# Supplementary material for: The impact of short-term reduction in hepatic triglycerides on vascular health in adults with or at risk of metabolic dysfunction-associated steatotic liver disease
Source: PLoS One. 2026 Aug 13;21(8):e0345338. doi: 10.1371/journal.pone.0345338 (PMC13472412; doi:10.1371/journal.pone.0345338)
Supplement: Supplementary file 6 [file pone.0345338.s006.docx]

**S5 Table. Pearson correlation between changes in hepatic triglycerides and heart rate, brachial ankle pulse wave velocity and flow-mediated dilation in people with MASLD.**

|  | ∆ Hepatic triglycerides | ∆ Heart rate | ∆ Brachial-ankle PWV | ∆ Flow-mediated dilation |
| --- | --- | --- | --- | --- |
| ∆ Hepatic triglycerides | 1 | *r* = 0.268 (*p* = 0.152) | *r* = -0.132 (*p* = 0.486) | *r* = 0.272 (*p* = 0.189) |
| ∆ Heart rate | *r* = 0.268 (*p* = 0.152) | 1 | *r* = 0.017 (*p* = 0.926) | *r* = 0.150 (*p* = 0.464) |
| ∆ Brachial-ankle PWV | *r* = -0.132 (*p* = 0.486) | *r* = 0.017 (*p* = 0.926) | 1 | *r* = -0.087 (*p* = 0.673) |
| ∆ Flow-mediated dilation | *r* = 0.272 (*p* = 0.189) | *r* = 0.150 (*p* = 0.464) | *r* = -0.087 (*p* = 0.673) | 1 |
